# Magazines and Exchanges

**Published:** 1898-11

**Authors:** 


### *MAGAZINES AND EXCHANGES.*

---

*The Texas Clinic* is a new publication from the ranks of the medical profession in the Lone Star State. Its first issue is excellent, and we welcome it among our exchanges. It is published in Dallas, Texas, and Dr. J. B. Shelmire is its editor.

---

*Langsdale Lancet*, published in Kansas City, Mo., so favorably known for the last few years among our exchanges, announces in its September issue that beginning with the October number the name of the *Lancet* will be changed to *The Kansas City Lancet*, and will be edited by Dr. Jno. Punton, the former editor, Dr. Langsdale, retiring. The *Langsdale Lancet* has always been welcomed among our exchanges as a most excellent medical journal, and in the new change which it makes we wish it every success.

---

*The Ladies' Home Journal* for October is replete with good things. Among its contents there are "Twenty Bright Stories about Mark Twain," "'Royal Letters' from Napoleon, Queen

Victoria, Napoleon III., and Emperior William I." Besides these the practical things discussed for the benefit of home life is well worth the price of the *Journal*. It is certainly the publication for the "home fireside."

---

*Our Dumb Animals* is still welcomed among our exchanges and brings bright home fireside chit-chat.

---

WE are in receipt of the *New York Sunday Times*, with a beautifully illustrated supplement. This is a new feature of this wide-awake paper and will no doubt add to its popularity.

